# Age-induced changes in skeletal muscle mitochondrial DNA synthesis, quantity, and quality in genetically unique rats

**DOI:** 10.1007/s11357-024-01344-4

**Published:** 2024-09-23

**Authors:** Robert V. Musci, Jordan D. Fuqua, Frederick F. Peelor, Hoang Van Michelle Nguyen, Arlan Richardson, Solbie Choi, Benjamin F. Miller, Jonathan Wanagat

**Affiliations:** 1 Department of Health and Human Sciences, Frank R Seaver College of Science and Engineering, Loyola Marymount University, 1 LMU Dr., Los Angeles, CA 90045 USA; 2 Aging and Metabolism Research Program, Oklahoma Medical Research Foundation, Oklahoma City, OK USA; 3 Department of Nutritional Sciences, University of Oklahoma Health Sciences, Oklahoma City, OK USA; 4 Department of Biochemistry & Physiology, University of Oklahoma Health Sciences, Oklahoma City, OK USA; 5 Oklahoma City Veterans Affairs Medical Center, Oklahoma City, OK USA; 6 Department of Medicine, Division of Geriatrics, UCLA, Los Angeles, CA USA; 7 Veterans Administration Greater Los Angeles Healthcare System, Los Angeles, CA USA

**Keywords:** Aging, Mitochondrial DNA, Skeletal muscle, Rats, Mutation, Deuterium oxide

## Abstract

Mitochondrial genomic integrity is a key element of physiological processes and health. Changes in the half-life of the mitochondrial genome are implicated in the generation and accumulation of age-induced mitochondrial DNA (mtDNA) mutations, which are implicated in skeletal muscle aging and sarcopenia. There are conflicting data on the half-life of mtDNA, and there is limited information on how aging affects half-life in skeletal muscle. We hypothesized that skeletal muscle mtDNA synthesis rates would decrease with age in both female and male rats concomitant with changes in mtDNA integrity reflected in mtDNA copy number and mutation frequency. We measured mitochondrial genome half-life using stable isotope labeling over a period of 14 days and assessed mtDNA copy number and deletion mutation frequency using digital PCR in the quadriceps muscle of 9-month-old and 26-month-old male and female OKC-HET rats. We found a significant age-related increase in mtDNA half-life, from 132 days at 9 months to 216 days at 26 months of age in OKC-HET quadriceps. Concomitant with the increase in mtDNA half-life, we found an age-related increase in mtDNA deletion mutation frequency in both male and female rats. Notably, 26-month-old female rats had a lower mutation frequency than male rats, and there were no changes in mtDNA copy number with sex, age, or mitochondrial genotype. These data reveal several key findings: (1) mtDNA turnover in rat skeletal muscle decreases with age, (2) mtDNA half-lives in skeletal muscle are approximately an order of magnitude longer than what is reported for other tissues, and (3) muscle mtDNA turnover differs significantly from the turnover of other mitochondrial macromolecules including components of the mitochondrial nucleoid. These findings provide insight into the factors driving age-induced mtDNA mutation accumulation, which contribute to losses of mitochondrial genomic integrity and may play a role in skeletal muscle dysfunction.

**Supplementary Information:**

The online version contains supplementary material available at 10.1007/s11357-024-01344-4.

## Introduction

The genome is essential for an organism’s development and function throughout its lifespan, and maintaining genome integrity is key. The maintenance of genome integrity encompasses dynamic processes such as DNA replication, repair, and turnover [1, 2]. Conversely, a loss of genome integrity with aging results from disruptions of these processes and leads to copy number variations, increased rates of DNA damage, and somatic mutation accumulation that contribute to phenotypes of aging [3–5]. Therefore, preserving genome integrity is a vital component of healthy aging.

While most studies of genome integrity focus on the nuclear genome, mitochondrial genome integrity is also impacted by aging and is linked to aging phenotypes [6]. Similar to the nuclear genome, age-induced changes in mitochondrial genome integrity include copy number variation, nucleotide damage, and mutations [7–10] that are linked to numerous age-related diseases [11–15]. A clear understanding of mitochondrial DNA (mtDNA) maintenance is needed to develop interventions against age-induced mtDNA integrity losses.

Distinctive features of mtDNA have a direct impact on its integrity. A given cell has hundreds of mtDNA copies, which are replicated and degraded independent of cell division [16]. The multicopy nature of mtDNA necessitates a high replicative index as compared to nuclear DNA leading to replication errors. Located on the inner mitochondrial membrane, mtDNA is prone to oxidative DNA damage and mutation due to electron transport [17]. Lastly, mtDNA lacks protective histone proteins and is missing some of the DNA repair pathways found in the nucleus [18]. Because these characteristics negatively impact mtDNA integrity, it is imperative to understand mtDNA maintenance, including synthesis and turnover.

Factors predisposing skeletal muscle to mtDNA defects include high energy demand, high mtDNA content to meet the energy demand, exposure to resulting oxidative stress, and the lack of cell division in myofibers to replenish mitochondria [19]. Many of these factors are dysregulated during muscle aging with resulting disruptions of mtDNA integrity. In aging mammalian skeletal muscle, mtDNA copy number declines [12, 20, 21] and mtDNA deletion mutations increase exponentially [12, 20, 22]. These age-induced changes differ between females and males; aged females have both lower mtDNA copy and mutation frequency than aged males in quadriceps muscle [12].

Turnover of mtDNA is one possible mechanism to maintain integrity. There are striking differences in reports of in vivo mtDNA half-life ranging from 9 to 350 days depending on the tissue and experimental approach. In adult rats, replicative tissues such as the liver [23] and adrenals [24] have mtDNA half-lives of 9 and 11 days, respectively. Tissues with less cell turnover have longer mtDNA half-lives. For example, mtDNA in the rat brain has a half-life of 31 days [25]. The experimental approach used to measure mtDNA half-life affects the observed values. Cardiac mtDNA half-life measured with ^3^H-thymidine is 6.7 days [23] but 350 days when measured using a stable isotope approach in the same Sprague–Dawley rat [26]. There have been no reports on the effect of aging on mtDNA half-life.

mtDNA half-life is a critical element in models of mtDNA mutation accumulation. In stochastic mathematical models, mtDNA turnover contributes significantly to the accumulation of mtDNA deletions [27–29]. Notably, these models assumed an mtDNA half-life of 1–10 days. However, stochastic modeling that assumes a half-life of 10 days predicted a much larger mtDNA deletion load than what is observed in vivo [30]. Instead, an mtDNA half-life of 300 days is better aligned with in vivo mtDNA mutation data [30]. The importance of mtDNA half-life in stochastic modeling of mtDNA deletion mutation accumulation and the discrepancy between models and in vivo findings suggest that these models may benefit from re-examining mtDNA half-life using stable isotope approaches and determining if mtDNA half-life changes with age.

The goal of this study was to assess age-related differences in mtDNA half-life in skeletal muscle by measuring synthesis over 14 days. We hypothesized that mtDNA half-lives would be longer in aged animals compared to adult counterparts and that the longer half-lives would differ by sex and correlate with changes in mtDNA integrity as assessed by mtDNA copy number and mutation frequency. We found that mtDNA half-life increased with age and mirrors the increase in mtDNA deletion mutation frequency but was not affected by sex at these ages. These findings hold implications for the formation and accumulation of mtDNA mutations, as well as the dynamics of mtDNA turnover with aging.

## Materials and methods

### Animal husbandry and stable isotope labeling

**Fig. 1:**
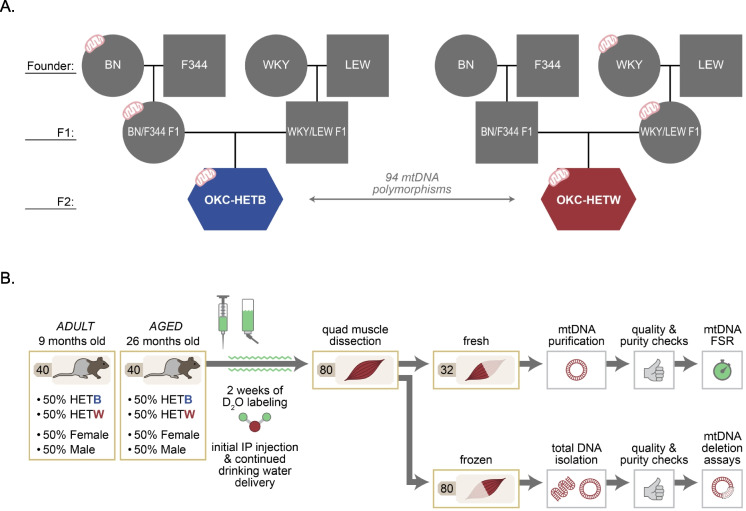
OKC-HET rat model breeding scheme and experimental design. **A** Breeding scheme and mtDNA genotype for each cross in the OKC-HET rat model. BN, Brown-Norway; F344, Fischer 344; WKY, Wistar-Kyoto; LEW, Lewis. **B** Experimental design for mtDNA FSR and mtDNA molecular measures

The study was carried out in accordance with the recommendations in the NIH Guide for Care and Use of Laboratory Animals. The protocols used were approved by the Institutional Animal Care and Use Committee at the Oklahoma City VA. Male (*n* = 20 for each age) and female (*n* = 20 for each age) OKC-HET rats were aged for 9 or 26 months and had free access to water and food. We included 9-month-old and 26-month-old animals to represent adult and aged male and female rats. The rats were generated by crossing Brown Norway, Fischer 344, Lewis, and Wistar-Kyoto rats (Fig. 1A) [31]. OKC-HET rats were created from reciprocal crosses between males and females from these two F1 hybrids. Half of the F2 rats have the BN mitochondrial genotype (OKC-HETB), and the other half have the WKY mitochondrial genotype (OKC-HETW). Rats were housed on a 14:10-h light/dark cycle in a 27 °C and humidity-controlled rodent facility. To assess fast- and slow-turning over mtDNA, we administered the stable isotope deuterium oxide (D_2_O) over the course of 14 days. Rats received an intraperitoneal injection of D_2_O-enriched (99%) sterile saline equivalent to 5% of the body water pool 14 days before euthanasia and harvest of tissues. For the 14 days, the rats drank water enriched with deuterium (8%) to maintain body water enrichment. This approach measures the average rate of turnover over 14 days but does not describe the specific turnover of rate of different mtDNA subpopulations that have differing half-lives.

### Tissue collection and processing

Prior to the end of the labeling period, one female rat in the 26-month-old cohort died unexpectedly for a final sample size of 79. Animals were anesthetized under isoflurane and euthanized by exsanguination. Serum acquired from the cardiac puncture was stored at − 80 °C for body water enrichment determination. Bone marrow was isolated from the tibia to determine the deuterium enrichment of a fully turned-over DNA pool. Quadriceps muscles were dissected, and half of one quadriceps muscle was used for mtDNA isolation and the other half was flash frozen in liquid nitrogen for total DNA isolation at a later time. Frozen muscle samples were stored at − 80 °C until analyzed. We chose to focus on the quadriceps muscle, as this proximal muscle shows marked age-related changes and is a mixed fiber type that we have characterized extensively in the aging F344 x BN F1 hybrid strain [20, 21, 32–35]. The fresh muscle samples used for mtDNA isolation were taken from the first four rats of each tissue harvest session to accommodate the 6 h required for the isolation procedure.

### mtDNA purification, quality control, and assessment of purity

mtDNA was purified from fresh quadriceps muscles using a published approach [26, 36]. Briefly, one-half of a fresh quadriceps muscle was minced, briefly digested with proteinase, and then mitochondria isolated by differential centrifugation. The isolated mitochondria are incubated with DNase I to digest nuclear DNA that is outside of the mitochondria while the mtDNA is protected inside the mitochondrial matrix. The digested nDNA is then washed away, and the mtDNA is purified from the mitochondrial pellet as using GenFind V3 (Beckman Coulter). mtDNA quality and quantity were assessed using spectrophotometry at A230, A260, and A280 (Thermo Scientific Nanodrop 2000 Spectrophotometer), fluorometry (Thermo Fisher Qubit 2.0 Fluorometer), and integrity examined by gel electrophoresis or TapeStation 4200 (Agilent). Measurement and reporting of mtDNA purity are essential because nuclear DNA contamination of the DNA sample will affect the fractional synthesis rate (FSR). Nuclear DNA in muscle homogenates proliferates at a slower rate than the reported values of mtDNA [37]. For example, myonuclei, which were presumed to be post-mitotic, proliferate at a rate of 2.5–8% per year [38]. Contamination of mtDNA samples with nuclear DNA would result in lower FSRs and longer half-lives. mtDNA purity in our study was assessed using quantitative PCR using nDNA and mtDNA-specific primer/probe sets as previously described [20]. The copy number ratio of mtDNA to nDNA was converted to a mass ratio for reporting the percent mass of mtDNA in each sample.

### mtDNA copy number and deletion frequency analyses

mtDNA copy number and deletion mutation frequency were measured using previously validated digital PCR approaches [20]. The other half of the quadriceps muscle that was used for the mtDNA isolation was frozen in liquid nitrogen. The frozen quadriceps muscle was powdered using a mortar and pestle under liquid nitrogen. Total DNA was extracted from 25 mg of muscle powder using GenFind V3 (Beckman Coulter). Total DNA was eluted or resuspended in 10 mM Tris–EDTA buffer, pH 8. Total DNA quality and quantity were assessed using spectrophotometry at A230, A260, and A280 (Thermo Scientific Nanodrop 2000 Spectrophotometer), fluorometry (Thermo Fisher Qubit 2.0 Fluorometer), and integrity examined by gel electrophoresis or TapeStation 4200 (Agilent).

A 5-prime nuclease cleavage assay and droplet-based digital PCR (ddPCR) were used to quantitate copy numbers for nuclear DNA, total mtDNA, and mtDNA deletion mutations with specific primer/probe sets for each as previously described [20]. Samples were diluted to the manufacturer’s recommended target range (20 to 2000 target copies per microliter). Digital PCR cycling conditions were polymerase activation at 95 °C for 10 min, 40 cycles of denaturation at 94 °C for 30 s, and annealing/extension at 60 °C for 2 min. Reaction threshold and target copy number per microliter were determined using QuantStudio 3D Analysis Suite Cloud Software (Version 3, Thermo Fisher; Waltham, MA). Direct quantitation of the major arc deletions by dPCR used the same cycling conditions but for 60 cycles. Researchers performing the molecular assays were blinded to sample characteristics.

### mtDNA isotope measurements

We measured deuterium oxide enrichment of mtDNA and total bone marrow DNA. Isolated DNA from bone marrow and purified mtDNA from the quadriceps were analyzed for deuterium enrichment as we have previously described [38–40]. Isolated DNA and mtDNA were hydrolyzed at 37C with nuclease S1 and potato acid phosphatase. Hydrolysates were reacted with nuclease S1 and potato acid phosphatase. Hydrolysates were reacted with pentafluorobenzyl hydroxylamine and acetic acid and then acetylated with acetic anhydride and 1-methylimidazole. Dichloromethane extracts were dried, resuspended in ethyl acetate, and analyzed by GC–QQQ (Agilent 8890 GC coupled to Agilent 7010B GC-QQQ, Santa Clara, CA, USA) on a DB-17 column with negative chemical ionization, using helium as a carrier and methane as the reagent gas. The fractional molar isotope abundances at m/z 435 (M0, the mass isotopomer) and 436 (M1) of the pentafluorobenzyl triacetyl derivative of purine deoxyribose were quantified using ChemStation software.

We calculated excess fractional *M* + 1 enrichment of the sample (EM1) as the amount of relative enrichment beyond background levels of enrichment using unenriched pentafluorobenzyl triacetyl purine dR derivative standard as follows:

1 $$\mathrm{EM}1 = \frac{\left(\mathrm{abundance} \frac{m}{z}436\right)\mathrm{sample}}{\left(\mathrm{abundance} \frac{m}{z}\mathrm{435,436}\right)\mathrm{sample}}- \frac{\left(\mathrm{abundance} \frac{m}{z}436\right)\mathrm{standard}}{\left(\mathrm{abundance} \frac{m}{z}\mathrm{435,436}\right)\mathrm{standard}}$$

The fraction new of mtDNA over 14 days was calculated as the enrichment (EM1) of mtDNA (product) divided by either plasma enrichment or bone marrow DNA enrichment. Over the 2-week labeling period, the DNA of bone marrow fully renews; thus, the enrichment is equal to the true precursor enrichment. In some cases where bone marrow could not be used, plasma enrichment with mass isotope distribution adjustment (MIDA) was used to estimate precursor.

2 $$\text{Fraction new }= \frac{\mathrm{product}}{\mathrm{precursor}}$$

Fractional synthesis rate (FSR) was calculated by dividing the fraction new by the duration of label (i.e., 14 days).

3 $$\mathrm{FSR }= \frac{\text{fraction new}}{\text{label duration}}$$

We calculated half-life (*t*_1/2_) by first calculating the rate parameter *k* as follows:

4 $$k =-\mathrm{ln}\left(\frac{1-\text{fraction new}}{\text{label duration}}\right)$$

To determine half-life (*t*_1/2_), we assumed mtDNA turnover is a first-order reaction where turnover is in a steady state and the rate constant is the natural log of 2 (i.e., 0.693) and performed the following calculation:

5 $${t}_\frac{1}{2}=\frac{0.693}{k}$$

### Statistical analysis

All data were tested for normal distribution. Data with normal distribution were presented as means ± SEM. A three-way ANOVA (age × sex × genotype) was used to compare differences among multiple groups. However, we found no difference in mtDNA copy number or mutation frequency between OKC-HETB and OKC-HETW strains. Thus, we collapsed the genotype groups and proceeded with using two-way ANOVA (age × sex) with Tukey post hoc analysis for multiple comparisons to describe differences between groups. Pearson correlation tests were conducted to determine the relationship between quadriceps mtDNA copy number or deletion mutation frequency with the muscle masses of the gastrocnemius, plantaris, soleus, tibialis anterior, and extensor digitorum longus. We used Levene’s test to test for differences between the variability of groups.

## Results

### Purity of the enriched mtDNA isolate affects fractional synthesis rates

**Fig. 2:**
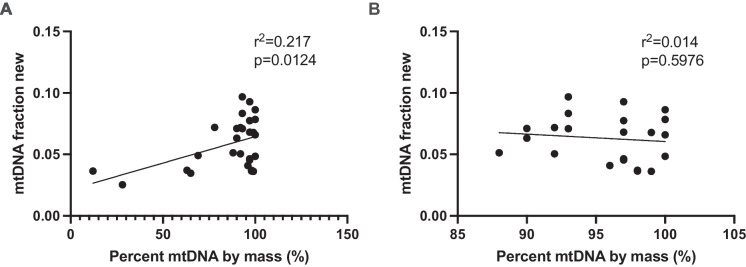
Nuclear DNA contamination affects measured D_2_O enrichment of enriched mtDNA. Over a wide range of purity levels, there is a weak correlation between mtDNA purity and measured mtDNA fraction new (**A**). Using only samples with a purity of greater than 85% mtDNA by mass, no relationship was observed between purity and mtDNA fraction new (**B**). Data were analyzed as a simple linear regression

In a subset of 28 quadriceps samples from a total of 79 rats, we measured mtDNA FSR using the stable isotope deuterium oxide. First, we measured the mtDNA purity of the nucleic acid samples by qPCR. We found that the mtDNA purity of the nucleic acid samples ranged from 12 to 100% mtDNA by mass (Fig. 2A). Six samples with complications during tissue homogenization had the most nuclear DNA contamination ranging from 16% nDNA to 88% nDNA by mass. Contamination with nuclear DNA correlated weakly with a decline in fraction new (*r*^2^ = 0.217, *p* = 0.0124, Fig. 2A). The exclusion of samples with mtDNA purity less than 85% abrogated this correlation (*r*^2^ = 0.014, *p* = 0.598, Fig. 2B). For subsequent fractional synthesis rate and half-life analyses, we excluded samples that had complications during the tissue homogenization as these had an mtDNA purity of less than 85%. The resulting set of 22 samples had an average purity of 96 ± 1% mtDNA by mass.

### mtDNA synthesis rates affected by age but not sex in OKC-HET rats

**Fig. 3:**
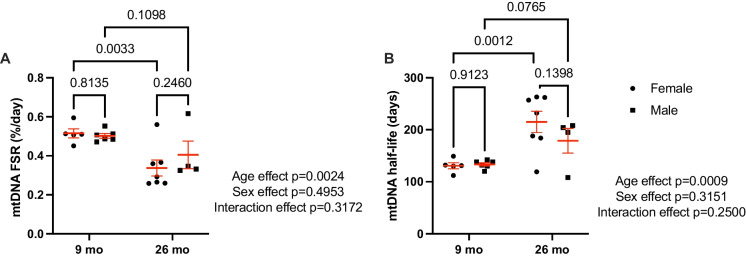
mtDNA half-life is longer in aged OKC-HET rats. mtDNA FSR (**A**) is slower and half-life (**B**) is longer in 26-month-old OKC-HET rats compared to 9-month-old counterparts. *N* = 4–7 for each age and sex. The statistical method used was two-way ANOVA with rat age and sex as factors

On average, quadriceps mtDNA FSR was lower in 26-month-old as compared to the 9-month-old rats (main effect of age *p* = 0.0024) (Fig. 3A). The age effect persisted when animals were separated by sex. Twenty-six-month-old female rats had slower mtDNA synthesis rates than 9-month-old female rats (9 months: 0.515 ± 0.051%/day, 26 months: 0.338 ± 0.108%/day; *p* = 0.003). Twenty-six-month-old male rats also had slower mtDNA synthesis rates when compared to 9-month-old male rats (9 months: 0.502 ± 0.029%/day, 26 months: 0.405 ± 0.141%/day; *p* = 0.110). The slower synthesis rates in the older rats translate to longer mtDNA half-lives in both male and female rats (effect of age *p* = 0.0009) (Fig. 3B). The calculated mtDNA half-life was longer (*p* = 0.001) in 26-month-old females (215.00 ± 20.40 days) compared to 9-month-old females (130.80 ± 5.86 days). mtDNA half-life was also longer (*p* = 0.077) in 26-month-old males (178.75 ± 23.74 days) compared to 9-month-old males (133.33 ± 3.21 days). Variability of mtDNA FSR and half-life measures were greater in the 26-month-old animals as compared to the young (*p* = 0.039, Levene’s test). There were no sex differences measured in mtDNA FSR at either age.

### Change in mtDNA FSR with age does not translate to change in mtDNA copy number between 9 and 26 months

**Fig. 4:**
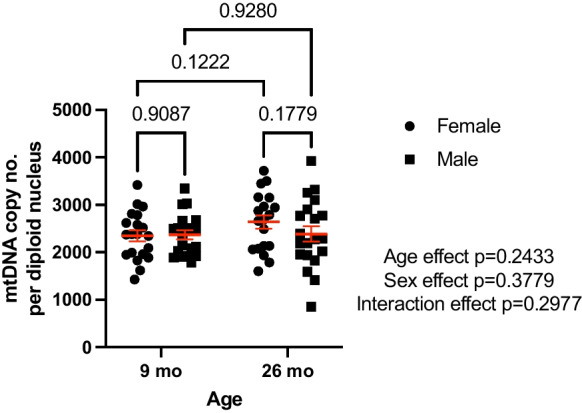
Quadriceps mtDNA copy number does not change with age in OKC-HET rats. Quadriceps mtDNA copy number is not different with sex or with age at 9 or 26 months in the OKC-HET rat. *N* = 19–20 for each age and sex. The statistical method used was a two-way ANOVA with rat age and sex as factors

The age-induced change in FSR led us to measure mtDNA copy number from total DNA samples extracted from the quadriceps muscle from the full set of 79 aging OKC-HET rats. We did not observe mtDNA copy number differences between the different mtDNA genotypes of the OKC-HETB and OKC-HETW strains in the quadriceps (Fig. S1) and thus collapsed genotypes for further analysis. There were no significant effects of either sex (*p* = 0.3779) or age (*p* = 0.2433) on mtDNA copy number (Fig. 4). mtDNA copy number in 9-month-old females was 2347 ± 113 versus 2368 ± 97 in the males (*p* = 0.9087), and copy number did not change at 26 months of age in either sex (2638 ± 141 in females and 2385 ± 163 in males; *p* = 0.1779). There was more variability in mtDNA copy number in the aged samples, most prominently in the 26-month-old male rats that ranged from 853 to 3924 copies per diploid nucleus (*p* = 0.083, Levene’s test).

Combining the mtDNA FSR data with the mtDNA copy number data, we were able to calculate the number of mitochondrial genomes being synthesized each day. In both male and female 9-month-old rat quadriceps, 12.7 ± 2.58 copies of mtDNA were being replicated out of ~ 2500 mtDNA copies per nucleus per day (Table S1). The copy number of replicated mtDNA significantly (*p* = 0.0003) decreases by ~ 37.8% to 7.89 ± 2.23 copies of mtDNA per nucleus synthesized per day in 26-month-old rats.

### Quadriceps muscle mtDNA copy number predicts the mass of lower hindlimb muscle in 26-month-old OKC-HET male but not female rats

**Fig. 5:**
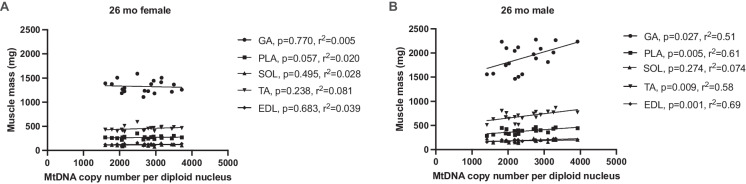
Correlation between quadriceps mtDNA copy number and other muscle masses. Quadriceps mtDNA copy number is related to other hindlimb muscle masses in 26-month-old male OKC-HET only. *N* = 18–19 for each age and muscle. Abbreviations: GA, gastrocnemius; PLA, plantaris; SOL, soleus; TA, tibialis anterior; EDL, extensor digitorum longus. Data were analyzed as simple linear regressions

We did not have the mass of each quadriceps muscle due to constraints during the dissection, but weights were measured for lower hindlimb muscles including the gastrocnemius (GA), plantaris (PLA), soleus (SOL), tibialis anterior (TA), and extensor digitorum longus (EDL). In 26-month-old females, quadriceps mtDNA copy number did not correlate with the masses of any of the lower hindlimb muscles (Fig. 5A). In 26-month-old male OKC-HET rats, the mtDNA copy number in the quadriceps predicts muscle mass in the GA, PLA, TA, and EDL, with coefficient of determination (*r*^2^) values > 0.50, but not the SOL (Fig. 5B). In 9-month-old rats, there were no significant correlations between quadriceps mtDNA copy number and lower extremity masses in females, while in the males, only the PLA had a significant negative correlation (Fig. S2).

### mtDNA deletion mutation frequency is higher with age and in male OKC-HET rats

**Fig. 6:**
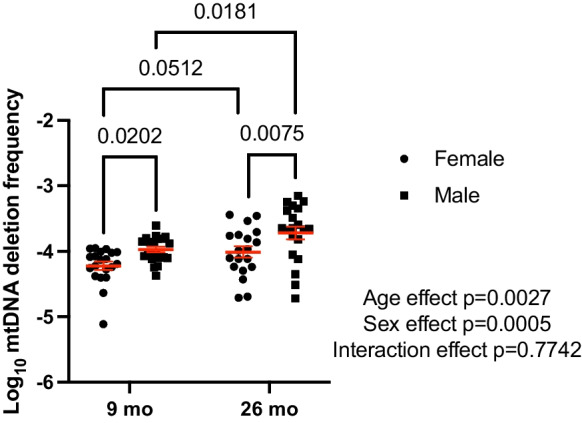
mtDNA deletion mutation frequency is greater in aged OKC-HET rats. mtDNA deletion mutation frequency on a logarithmic scale is greater in male compared to female OKC-HET rats and is greater in 26-month-old compared to 9-month-old rats. *N* = 19–20 for each age and sex. The statistical method used was two-way ANOVA with rat age and sex as factors

In the same total DNA samples from which we measured mtDNA copy number, we did not observe mtDNA mutation frequency differences between the different mtDNA genotypes of the OKC-HETB and OKC-HETW strains in the quadriceps (*p* = 0.445, Fig. S3). We observed that mtDNA deletion frequency increased with age (main effect of age *p* = 0.0027) (Fig. 6). In female rats, mtDNA deletion frequency increased from 6.9*e* − 5 ± 6.67*e* − 6 at 9-month-old to 1.31*e* − 4 ± 2.39*e* − 5 at 26 months of age (*p* = 0.0512). Females had a lower mutation frequency than the males at each age (main effect of sex *p* = 0.0005). In males, mtDNA deletion frequency increased from 1.17*e* − 004 ± 1.14*e* − 005 at 9 months of age to 2.74*e* − 004 ± 4.48*e* − 005 at 26 months of age (*p* = 0.0181). As noted with copy number, the variability of mutation frequency increased with age in the male rats (*p* = 0.047, Levene’s test). Quadriceps mtDNA deletion frequency did not correlate with lower hindlimb muscle masses in males or females at 26 months of age (Fig. S4).

## Discussion

This study is the first report of age-induced changes in mtDNA synthesis and, given that mtDNA copy number was not different with age, our data are also the first to demonstrate that overall mtDNA turnover declines with age. Our finding of mtDNA half-lives in skeletal muscle ranging from 4 months in the adult rats to 7 months in the aged OKC-HET rats has implications on the accumulation of mtDNA mutations. We did not observe differences in quadriceps mtDNA copy number with age at these ages in the OKC-HET strain, but we did find that quadriceps mtDNA copy number predicts the mass of other muscles in aged male rats. Lastly, we found that quadriceps mtDNA deletion mutation frequency increases with age, more so in males than in females.

The data and findings in our study add to our current understanding of mtDNA integrity and aging. Since the late 1960s, more than a dozen studies across numerous species and tissues have measured mtDNA half-life [23–26, 41–46]. Most of these studies used short-term labeling with ^3^H-thymidine and only one measured mtDNA synthesis rates in skeletal muscle [26]. The majority of these studies did not verify the purity of the isolated mtDNA or report the amount of nDNA contamination. The previous study using D_2_O to measure mtDNA half-life in skeletal muscle was performed in 2–4-month and 8–10-month Sprague–Dawley rats [26]. That study measured an mtDNA half-life of 24 months in “hindlimb” muscle that differs considerably from our average half-life of 4 months in 9 months OKC-HET quadriceps. We are unable to compare these results directly without knowing the identity of the hindlimb muscle used in the previous study.

Our measurements of mtDNA copy number and mutation frequency are consistent with previous reports. mtDNA copy number predicts human quadriceps mass [12], a relationship between mtDNA copy number and muscle mass that we have now identified in rats, albeit only in 26-month-old males. We previously found that skeletal muscle mtDNA deletion frequency increases with age in mice, non-human primates, humans, and other rat strains [19–22, 47, 48]. The current study is the first report of sex differences in mtDNA deletion mutation frequency in rats. These findings are comparable to observations that females have lower mtDNA deletion mutation frequencies in both mouse [21] and human [12] skeletal muscle.

The reported range of skeletal muscle mtDNA half-lives of 4 to 24 months has implications for many aspects of mitochondrial genomics and aging. mtDNA synthesis studies in rat skeletal muscle report half-lives that are an order of magnitude longer than mtDNA half-lives in other tissues (e.g., brain, kidney, liver, and adrenal glands). The differences in reported mtDNA half-lives may be due to either tissue differences in mtDNA half-life or differences between short-term labeling with ^3^H-thymidine (i.e., typically 1–2 days) and longer-term stable isotope labeling over weeks as we performed in the present study. The ^3^H-thymidine approach is a known result in several issues that confound measuring synthesis rates (e.g., induction of cell-cycle arrest, apoptosis, DNA damage, and inhibition of DNA synthesis) [49]. The labeling duration has a significant effect on the observed half-life if the incorporation of the tracer or turnover is non-linear and/or non-homogenous (e.g., if there are subpopulations of mitochondria or mtDNA). For example, short labeling periods are biased in favor of macromolecules with rapid turnover, even if they are less abundant [50]. Studies reporting mtDNA half-lives between 10 and 30 days used short-term (< 48 h) toxic, radioactive labels [23–25]. However, with the use of a stable, non-toxic isotope tracer, we measured cumulative mtDNA synthesis over 14 days and found that only 5–7% of mtDNA had been synthesized per week. The longer labeling periods used in stable isotope labeling allow measurement of these differing populations (i.e., those with rapid turnover and those with slower turnover) and offer a more integrated view of the entire population. Collins and colleagues demonstrated that mtDNA synthesis rates decreased as labeling period lengthened from 1 to 8 weeks (*r* =  − 0.73) [26]. Additional time-course studies of mtDNA synthesis rates will provide more insight into the dynamics of mtDNA subpopulations.

Skeletal muscle-specific mtDNA synthesis rates and the age-induced decline in these rates will be of great use in modeling studies of mtDNA mutation accumulation [29, 51–54]. Each of these reports assumed an mtDNA half-life of 10 days based on the earlier ^3^H-thymidine studies. Because mtDNA half-life is a key element of mutation accumulation models, the models will need to account for muscle-specific synthesis rates in the range of 4 to 24 months as well as the age-induced declines in mtDNA synthesis [54].

Our data show that mtDNA may be one of the longest-lived mitochondrial components in skeletal muscle mitochondria. Mitochondria contain a variety of macromolecules, including proteins, lipids, and nucleic acids, each with distinct ranges of half-lives. Employing stable isotopes, we have demonstrated that skeletal muscle mitochondrial protein half-lives range from hours to months [55] and, in skeletal muscle, on average, mitochondrial proteins are longer lived than cytoplasmic proteins [56–58]. In the 5–8-month-old mouse soleus muscle, mitochondrial proteins have a median half-life of 22.3 days versus a mean half-life of 16.4 days for cytoplasmic proteins. In cardiac muscle, the mean half-life of muscle mitochondrial lipids is also much shorter than mtDNA with distinct pools of cardiolipin having half-lives as short as 3 days [59]. The observation that individual mitochondrial protein, lipid, and DNA turnover rates span at least two orders of magnitude suggests a complex landscape of mitochondrial turnover against a backdrop of processes including selective mitophagy, fission, and fusion.

We have begun to examine the use of mtDNA copy number and/or mutation frequency as markers of biological age and longevity [12, 21, 35, 48, 60]. The sex differences in the relationship of mtDNA copy number to muscle mass and the lower mtDNA deletion mutation frequency in female rat quadriceps suggest that female OKC-HET rats are biologically younger than males. Based on these observations, we predict that the females of the OKC-HET four-way cross will have a longer lifespan than the males. While the prediction that females will live longer than males may seem trivial, as female longevity holds true across many species including humans, it is worth noting that in some rat strains, males live longer than females. For instance, in the F344xBN F1 hybrid rat, widely used in aging research and supported by the National Institute on Aging, the mean lifespan for females is 30 months but 34 months for males [61]. The results of lifespan studies for OKC-HET rats will provide an intriguing test of mtDNA deletion mutations as a predictive biomarker of longevity.

There are a few limitations to our study. Based on our statistical analyses, we were underpowered to detect the effects of sex or mitochondrial genotype on mtDNA FSR. We only measured mtDNA FSR in a subset of animals due to the need for fresh tissue and a processing time of 6 h for four samples that limit the number of samples that may be processed in 1 day. Development and validation of methods for mtDNA isolation from frozen muscle and smaller amounts of muscle will allow access to a larger number of samples including stored samples. For this study, we used a labeling period of 14 days. Therefore, if there is a mtDNA subpopulation that is fully turned over before 14 days, our half-life values will not fully reflect this subpopulation. Without a time course of stable isotope labeling, we cannot know if we reached a plateau of stable isotope enrichment. There is evidence that mtDNA subpopulations turnover at different rates or not at all [26, 62]. It may be that there is a small population that turns over rapidly and a larger population with a much slower turnover. The current approach cannot distinguish half-lives of mutant or wildtype mtDNA or various subpopulations. Not accounting for a subpopulation of mtDNA that is resistant to turnover may profoundly affect the understanding of mechanisms related to mtDNA maintenance [63]. An additional matter particularly in measuring mtDNA turnover in bulk skeletal muscle tissue is the numerous mitochondrial subpopulations that exist within the skeletal muscle, including interfibrillar, paravascular, and subsarcolemmal mitochondria [64], and the various cell types within the skeletal muscle niche.

In conclusion, our findings demonstrate that quadriceps muscle mtDNA synthesis is slowed during aging and aligns with an earlier study of skeletal muscle mtDNA synthesis. The available data on muscle mtDNA synthesis suggest that muscle mtDNA turnover differs from other tissues and may be influenced by the existence of multiple mtDNA populations with differing turnover. The decrease in mtDNA turnover with aging is concomitant with increased mtDNA deletion mutation frequency, but more research is necessary to understand the mechanisms, if any, linking these phenomena. Future research should examine more closely the different mtDNA populations, which would require time-course studies and further study of the effects on mutation frequency of altering mtDNA half-life.

## Supplementary Information

Below is the link to the electronic supplementary material.

Supplementary file1 (DOCX 224 kb)

## Data Availability

All supporting data can be found in the paper and supplementary files. Additional original data can be provided upon request. Contact R.V.M. describing the specific data requested and the intended use of the data.
