# Supplementary material for: Age-induced changes in skeletal muscle mitochondrial DNA synthesis, quantity, and quality in genetically unique rats
Source: GeroScience. 2024 Sep 23;47(1):851–62. doi: 10.1007/s11357-024-01344-4 (PMC11872842; doi:10.1007/s11357-024-01344-4)
Supplement: Supplementary file 1 — Supplementary file1 (DOCX 224 kb) [file 11357_2024_1344_MOESM1_ESM.docx]

**Supplementary Table 1.** Summary table of rat strain, sex, and age and mtDNA purity, mtDNA fraction new, mtDNA copy number per diploid nucleus, and mtDNA synthesized per day of samples included in isotope analysis. Table includes all samples used for isotope analyses. Samples highlighted in red were excluded from analyses due to poor mtDNA purity (see Figure 2). There was a significant effect of Age (p=0.002) on mtDNA fraction new, but not Sex (p=0.495). In this cohort, just as in the complete sample, there was no effect of Age (p=0.533) or Sex (p=0.639) on mtDNA copy number. Assessing the number of new mtDNA synthesized per day, there was a significant effect of Age (p=0.0003), but not Sex (p=0.589).

**Supplementary Figure S1.** Quadriceps mtDNA copy number is not different between OKC-HETB and OKC-HETW genotypes. To verify that OKC-HET strain did not affect mtDNA copy number, we conducted a Three-Way ANOVA with Age, Sex, or Genotype as factors. There was no significant main effect of Age (p=0.276), Sex (p=0.362), or Genotype (p=0.754) on mtDNA copy number in the quadriceps. N= 10 for each Age, Sex, and Genotype, except for 26mo HETB females (N = 9), 26mo HETB males (N = 11), and 26mo HETW males (N = 9).

**Supplementary Figure S2.** Quadriceps mtDNA copy number is not related to lower extremity masses in 9mo male or female OKC-HET rats. There was no statistically significant (p<0.05) relationship between mtDNA copy number of quadriceps in 9mo female OKC-HET rats. There was a significant, inverse modest relationship (r^2^ = 0.206) between mtDNA copy number in the quadriceps and muscle mass of the plantaris in 9mo male OKC-HET rats. However, there were no other statistically significant (p<0.05) relationships in 9mo male OKC-HET rats. N=20 for each sex and muscle. Abbreviations: GA: gastrocnemius; PLA: plantaris; SOL: soleus; TA: tibialis anterior; EDL: extensor digitorum longus.

**Supplementary Figure S3.** Quadriceps mtDNA deletion mutation frequency is not different between OKC-HETB and OKC-HETW genotypes. To verify that OKC-HET strain did not affect mtDNA deletion mutation frequency, we conducted a Three-Way ANOVA with Age, Sex, or Genotype as factors. There was no significant main effect of Genotype (p=0.445) on mtDNA deletion mutation frequency in the quadriceps. Both Age (p=0.003) and Sex (p=0.0007) had a significant effect on mtDNA deletion mutation frequency. N=9 – 10 for each Age, Sex, and Genotype

**Supplementary Figure S4.** mtDNA deletion mutation frequency is not related to lower hindlimb extremity masses in 26mo male or female OKC-HET rats. There was no statistically significant (p<0.05) relationship between mtDNA deletion mutation frequency in the quadriceps and lower extremity muscle masses in 26mo male or female OKC-HET rats. N=18-19 for each age and muscle. Abbreviations: GA: gastrocnemius; PLA: plantaris; SOL: soleus; TA: tibialis anterior; EDL: extensor digitorum longus.

**Supplementary Table 1**

| **Sample ID** | **Strain** | **Age (mo)** | **Sex** | **Estimated mtDNA by mass (%)** | **mtDNA fraction new** | **mtDNA copy no.**  **(#/diploid nucleus)** | **Daily mtDNA synthesis (#/nucleus/per day)** |
| --- | --- | --- | --- | --- | --- | --- | --- |
| Y01 | HET W | 9 | F | 90 | 0.0632 | 2783.90 | 12.57 |
| Y02 | HET B | 9 | F | 78 | 0.0720 | *2965.83* | *15.25* |
| Y04 | HET W | 9 | F | 93 | 0.0833 | 2381.55 | 14.17 |
| Y11 | HET W | 9 | M | 97 | 0.0774 | 3344.42 | 18.49 |
| Y12 | HET B | 9 | M | 99 | 0.0678 | 3026.09 | 14.65 |
| Y13 | HET W | 9 | M | 28 | 0.0253 | *2470.25* | *4.46* |
| Y15 | HET B | 9 | M | 100 | 0.0660 | 2383.26 | 11.24 |
| Y21 | HET B | 9 | F | 97 | 0.0928 | 1618.81 | 10.73 |
| Y22 | HET W | 9 | F | 90 | 0.0711 | 1828.01 | 9.28 |
| Y23 | HET B | 9 | F | 69 | 0.0491 | *1960.56* | *6.88* |
| Y25 | HET W | 9 | F | 93 | 0.0968 | 1995.35 | 13.80 |
| Y31 | HET B | 9 | M | 92 | 0.0718 | 1921.26 | 9.85 |
| Y32 | HET W | 9 | M | 63 | 0.0371 | *1786.41* | *4.73* |
| Y33 | HET W | 9 | M | 93 | 0.0710 | 2508.57 | 12.72 |
| Y34 | HET W | 9 | M | 97 | 0.0681 | 2508.77 | 12.20 |
| O01 | HET B | 26 | M | 97 | 0.0455 | 2905.54 | 9.44 |
| O03 | HET B | 26 | M | 100 | 0.0863 | 1589.19 | 9.80 |
| O04 | HET W | 26 | M | 97 | 0.0465 | 2024.26 | 6.72 |
| O12 | HET W | 26 | F | 99 | 0.0362 | 2789.09 | 7.21 |
| O13 | HET B | 26 | F | 88 | 0.0513 | *2660.51* | *9.75* |
| O14 | HET W | 26 | F | 92 | 0.0504 | 3151.32 | 11.34 |
| O21 | HET W | 26 | M | 100 | 0.0485 | 1825.64 | 6.32 |
| O22 | HET B | 26 | M | 12 | 0.0365 | *2393.33* | *6.24* |
| O23 | HET W | 26 | M | 65 | 0.0347 | *3257.62* | *8.07* |
| O31 | HET W | 26 | F | 98 | 0.0371 | 2136.05 | 5.66 |
| O32 | HET B | 26 | F | 96 | 0.0410 | 2060.99 | 6.04 |
| O33 | HET W | 26 | F | 98 | 0.0364 | 2135.06 | 5.55 |
| O34 | HET W | 26 | F | 100 | 0.0785 | 1935.10 | 10.85 |

**Supplementary Figure S1**

**Supplementary Figure S2**

**Supplementary Figure S3**

**Supplementary Figure S4**
